# Supplementary material for: Intense pH Sensitivity Modulation in Carbon Nanotube-Based Field-Effect Transistor by Non-Covalent Polyfluorene Functionalization
Source: Nanomaterials (Basel). 2023 Mar 24;13(7):1157. doi: 10.3390/nano13071157 (PMC10096590; doi:10.3390/nano13071157)
Supplement: Supplementary file 1 [file nanomaterials-13-01157-s001.zip › nanomaterials-2279738-supplementary.pdf]

Supplementary Information

Intense pH sensitivity Modulation in Carbon Nanotube-Based Field-Effect Transistor By Non-Covalent Polyfluorene Functionalization

Gookbin Cho [1], Eva Grinenval [1], Jean-Christophe P. Gabriel [2] and Bérengère Lebental [3,*]

[1] Laboratoire de Physique des Interfaces et des Couches Minces, LPICM, CNRS, Ecole polytechnique, Institut Polytechnique Paris, 91128 Palaiseau, France
[2] Université Paris-Saclay, NIMBE (UMR CEA/CNRS 3685), LICSEN, 91191 Gif sur Yvette, France
[3] Université Gustave Eiffel, COSYS, IMSE, Marne-la-Vallée Campus, 77447 Marne-La-Vallée, France
* Correspondence: berengere.lebental@univ-eiffel.fr

1. Wirebonded chips on PCB

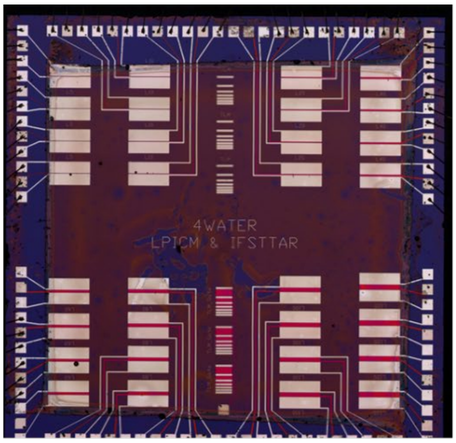

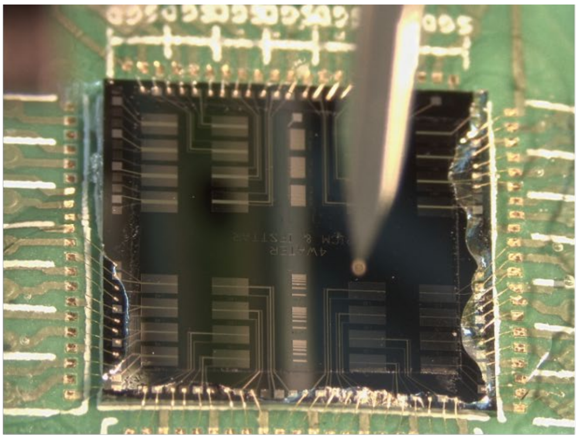

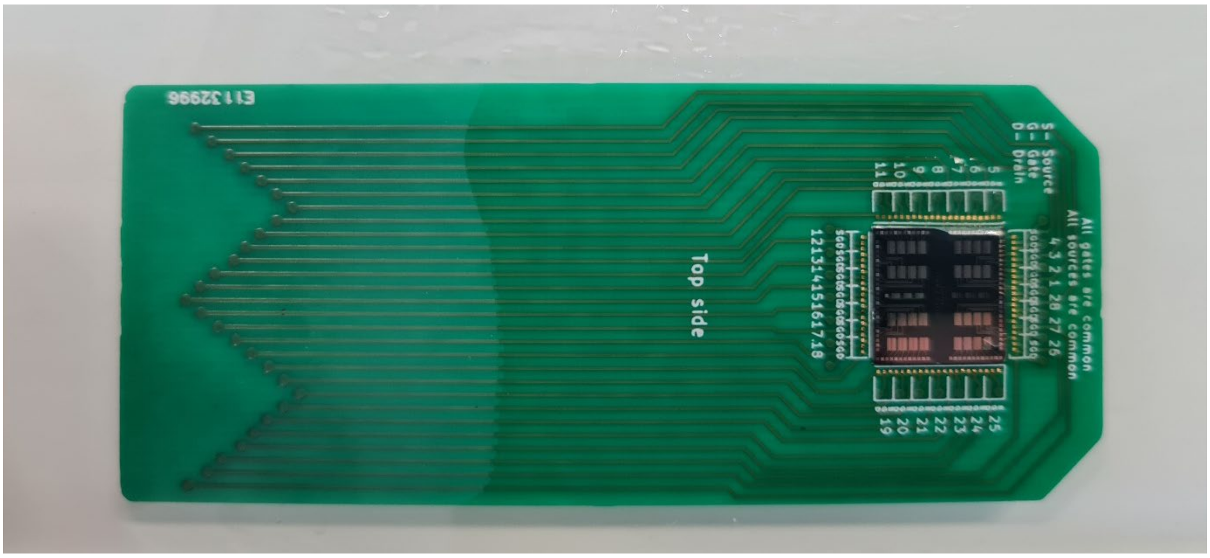

Figure S1. (upper-left) Ink-jet printed CNT-FET device after PMMA deposition process (upper-right) Wire bonded chip, with passivation of the wirebonding by UV resist. (below) Image of a PCB and CNT-FET devices after wire bonding and passivation process**.**

2. Formulation of the phosphate buffer

Table S1. Detailed amount of Sodium phosphate dibasic heptahydrate and Sodium phosphate monobasic monohydrate for 0.1 M phosphate buffer solution.

**pH**	**$Na_2HPO_4 \cdot 7H_2O$ (Sodium phosphate dibasic heptahydrate) (g)**	**$NaH_2PO_4 \cdot H_2O$ (Sodium phosphate monobasic monohydrate) (g)**	**Additional acid or base**
3	3.669	11.911	HCl until pH 3
4	3.669	11.911	HCl until pH 4
5	3.669	11.911	HCl until pH 5
6	3.669	11.911	
7	15.483	5.827	
8	15.483	5.827	NaOH until pH 8
9	15.483	5.827	NaOH until pH 9

3. Transfer curves in air

The electrical transfer curves of p-CNTFETs and f-CNTFETs in air before and after passivation were measured (Figure 2). They show hysteresis and charging effect for both types of CNTFETs, with both effects being particularly pronounced for the f-CNTFETs. As expected, passivation reduced the hysteresis and the charging effect.

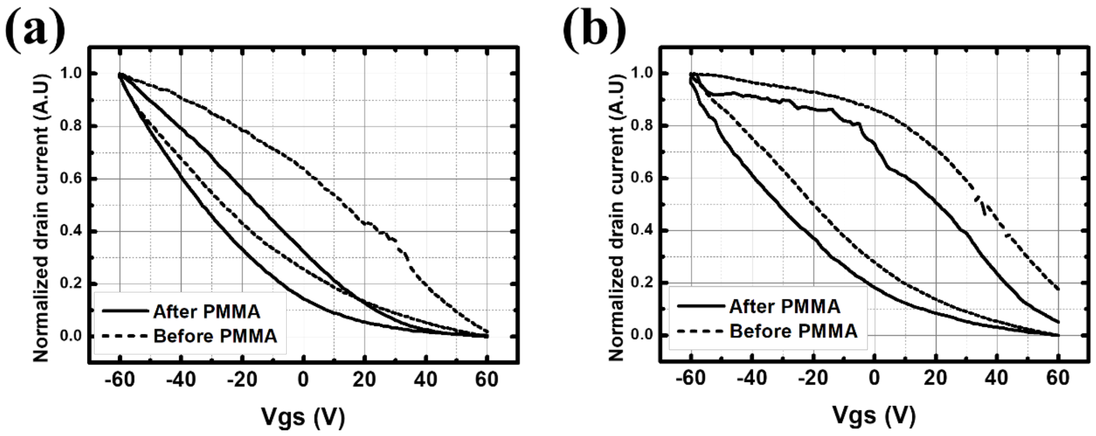

Figure S2. Normalized I-V transfer curve (e.g. I/I_{on} as a function of V_{gs}) of (a) p-CNTFET and (b) f-CNTFET before PMMA deposition (dotted line) and after PMMA deposition (sold line) in air. Channel length of each device is 5 µm.

Figure S3 shows the I_{on}/I_{off} ratio for the p-CNTFET as a function of channel length. It tends to increase up to 60 µm channel length and then stabilizes, with a possible outlier at 10 µm.

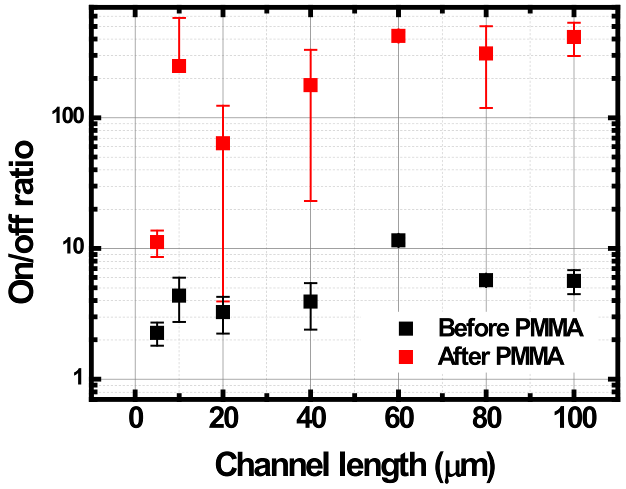

Figure S3. I_{on}/I_{off} ratio of p-CNTFETs before and after PMMA deposition with respect to channel length from 5 µm up to 100 µm.

Figure S4 and Figure S5 show I_{on} and I_{off} for p-CNTFET and f-CNTFET, respectively, as a function of channel length. The stronger effect of passivation on I_{off} than on I_{on} is clear. For p-CNTFET, the threshold channel length at 60 µm is more obvious than for I_{on}/I_{off} .

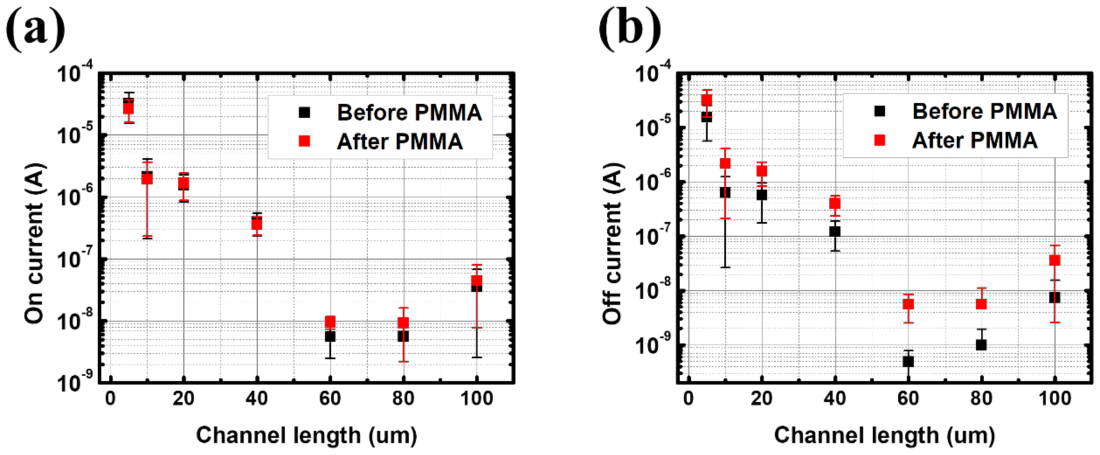

Figure S4. (**a**) I_{on} and (**b**) I_{off} of p-CNTFETs before and after PMMA passivation with respect to channel length from 5 µm to 100 µm.

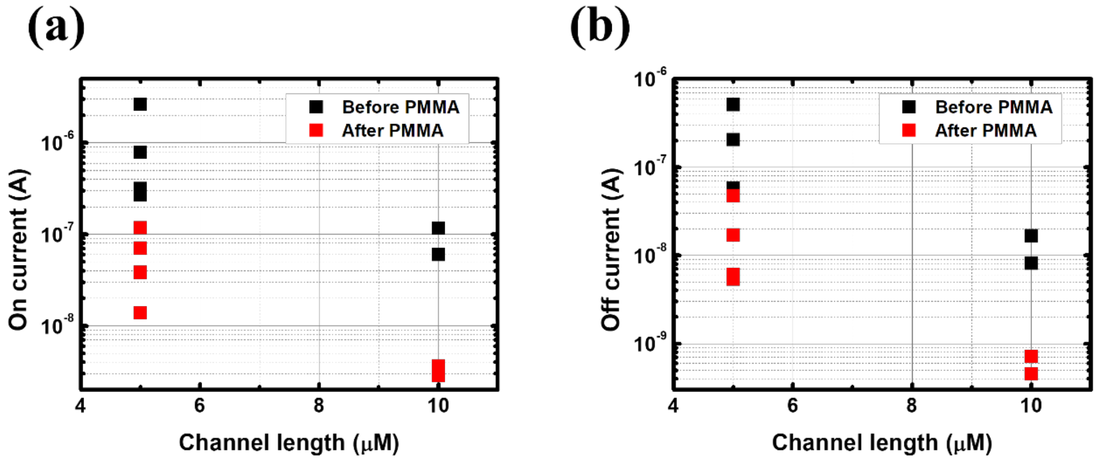

Figure S5. (**a**) I_{on} and (**b**) I_{off} of f-CNTFETs before and after PMMA passivation with respect to channel length from 5 µm to 10 µm.

4. CNTFET characterizations in water

Figure S6 shows the comparison of current levels in water at different gate voltages for a device without CNTs and a device with CNTs. The CNTFET clearly shows a transistor behavior in water between -1 V and +1 V. The comparison with the CNT-less chip shows that the transistor effect in water cannot be attributed to the conductivity of the water alone or to disturbances in the chip.

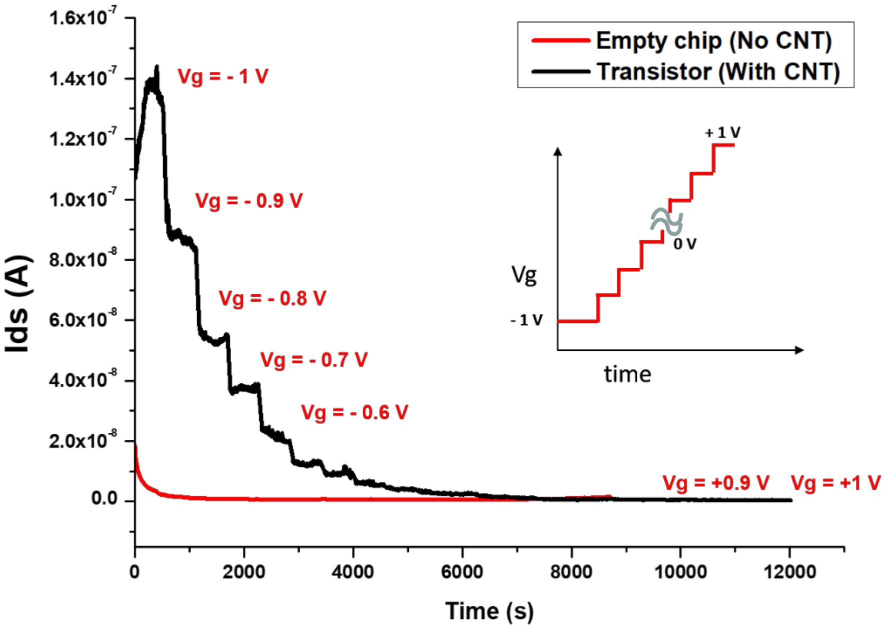

Figure S6. I-V transfer curves in water for p-CNTFET (Black solid line) and non-printed device (Red solid line) as reference.

Figure S7 shows the typical current response of a transistor over a pH step. To account for the stabilization time of the device after each chemical change, only the last 10% of the step is used to calculate the transistor response.

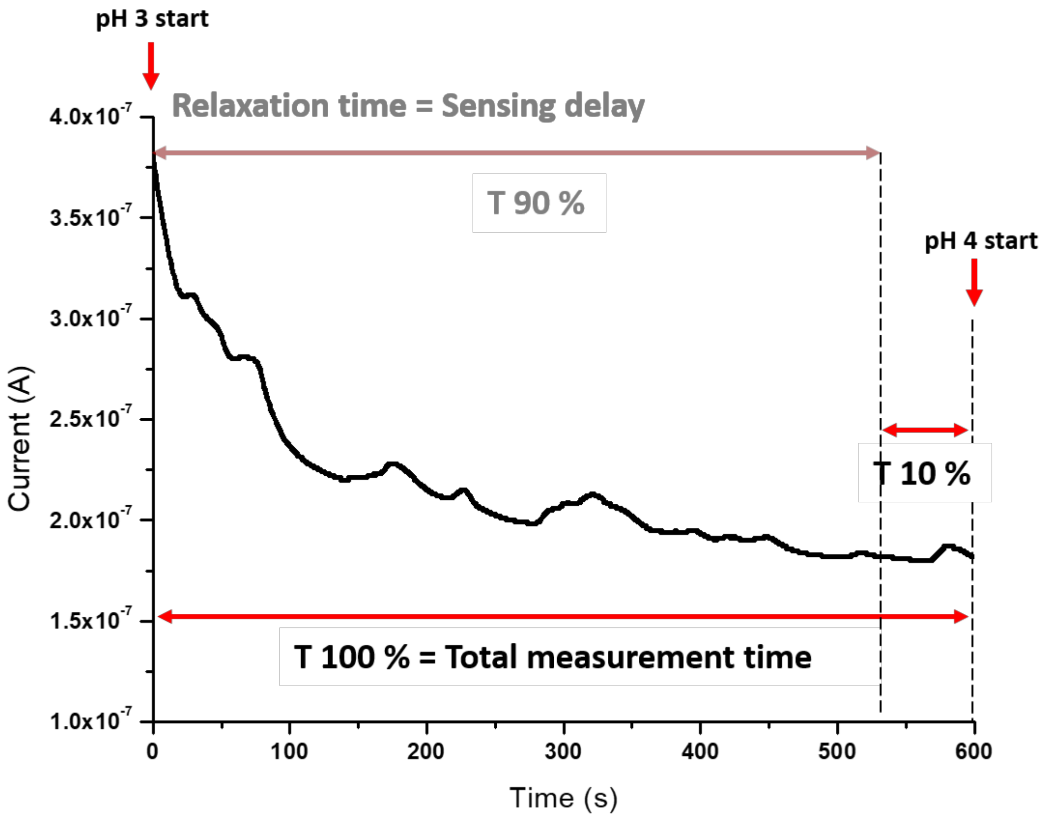

Figure S7. Data treatment process or real-time measured current values at a certain pH. The current response to a given pH step is calculated by averaging the last 10 percent of measured points for each pH step.

Figure S8 shows the I-V transfer curve of both types of CNTFETs in PBS at pH 7 in linear scale. The threshold voltage can be easily observed.

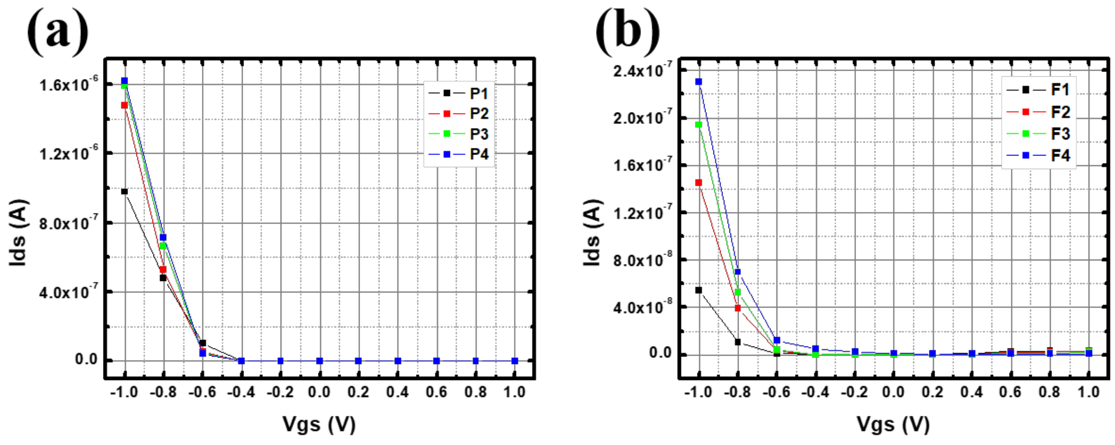

Figure S8. Linear I-V transfer curve of (a) p-CNTFETs and (b) f-CNTFETs in phosphate buffer solution (PBS) at pH 7. All transistors were 5 μm channel devices. V_{ds} was set to +0.8 V and V_{gs} was swept from −1 V to + 1 V.

5. I-V Transfer curves at different pH

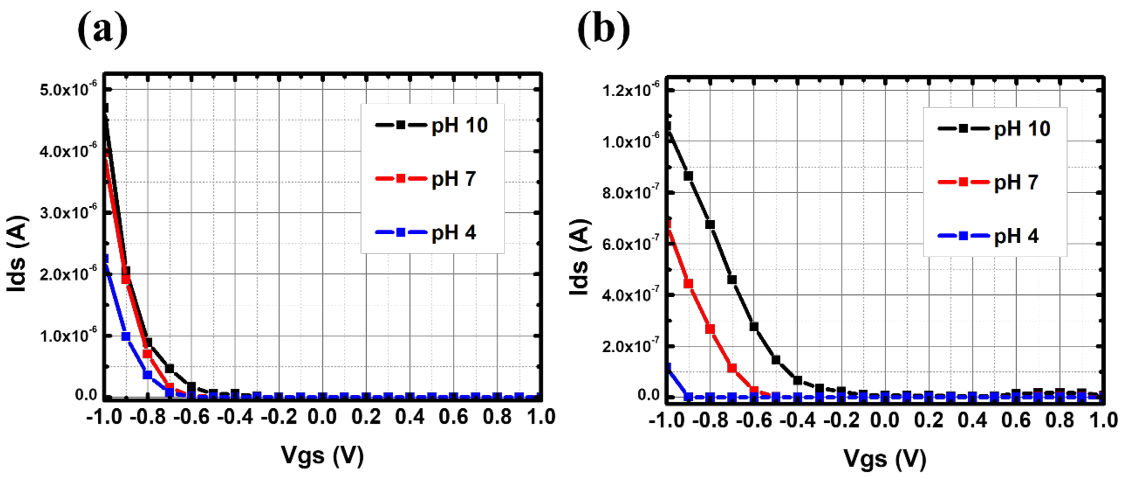

Figure S9. Linear I-V transfer curve of (a) p-CNTFETs and (b) f-CNTFETs in PBS with respect to different pH from pH 10 to pH 4. V_{ds} was fixed at + 0.8 V.

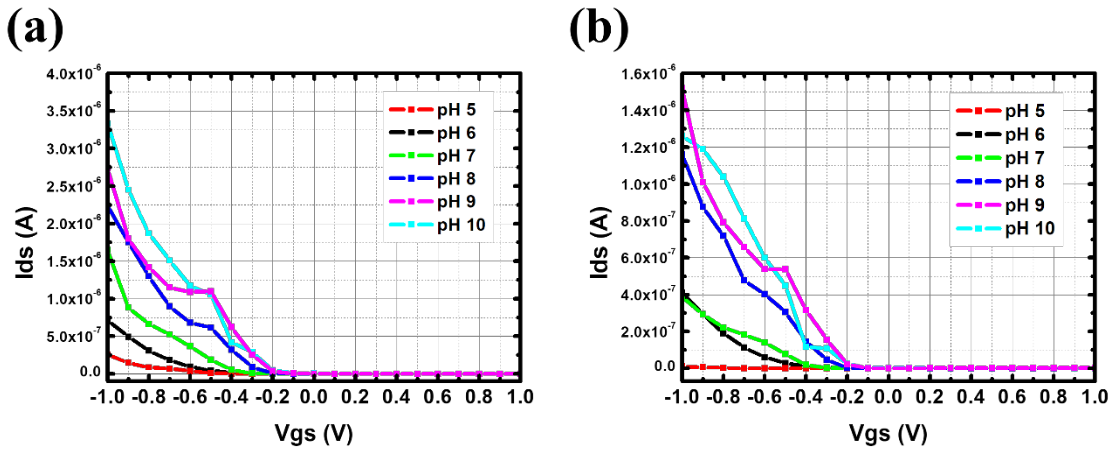

Figure S10. Linear I-V transfer curve of (a) p-CNTFETs and (b) f-CNTFETs in Borate buffer solution (BBS) with respect to different pH from pH 5 to pH 10. V_{ds} was fixed at + 0.8 V.
